# Metformin in Diabetic Retinopathy: Mechanisms, Therapeutic Potential, and Barriers

**DOI:** 10.7759/cureus.87455

**Published:** 2025-07-07

**Authors:** Hamza Alasbily, Fardous Ali Fahmi, Majdi Abdulhamid Abdala, Adnan Fouad Asheibi, Ahmed Adeil Amhidi, Ala Abed Elallegy, Adela H. Elamami

**Affiliations:** 1 Faculty of Dentistry, Department of Basic Medical Science, University of Benghazi, Benghazi, LBY; 2 Program of Basic Medical Science, Libyan International University, Benghazi, LBY; 3 Faculty of Medicine, Department of Ophthalmology, University of Benghazi, Benghazi, LBY; 4 Program of Medicine, Libyan International University, Benghazi, LBY; 5 Faculty of Medicine, Department of Internal Medicine, University of Benghazi, Benghazi, LBY

**Keywords:** ampk activation, diabetic retinopathy, metformin, microvascular complications, mitochondrial function, type 2 diabetes

## Abstract

The primary aim of diabetic retinopathy (DR) management is to prevent or slow the decline of vision loss. While current treatments for DR are effective in the advanced stages, they may not be as effective in the early phases of the disease. Metformin is among the most widely used antidiabetic medications and has recently shown promising outcomes in DR, as it has been found to affect several pathological mechanisms involved in DR, including oxidative stress, angiogenesis, and inflammation. Multiple clinical and preclinical studies demonstrated its potential role in reducing DR development and progression. However, the potential adverse effects, safety concerns, especially in patients with renal impairment, and the lack of large-scale studies supporting this evidence remain a barrier. This review explores the mechanisms through which metformin may exert its therapeutic effects in DR, discusses the clinical evidence, and identifies the limitations of metformin's potential use in DR treatment.

## Introduction and background

Among diabetes related-complications, diabetic retinopathy (DR) remains a common microvascular complication, inducing visual impairment in approximately 30-40% of patients at working age [1-3]. Several pathological mechanisms are linked to the development and progression of DR, with oxidative stress, inflammation, and angiogenesis being major contributors. Chronic hyperglycemia leads to excessive production of reactive oxygen species (ROS), surpassing endogenous antioxidant defenses, thereby inducing damage to retinal microvascular cells. This oxidative stress contributes to mitochondrial dysfunction, endothelial cell injury, and pericyte apoptosis, early events in DR pathogenesis. Additionally, the persistent hyperglycemic state facilitates the formation of advanced glycation end-products (AGEs), which interact with their receptors (RAGE) on retinal cells to amplify oxidative stress and pro-inflammatory signaling. The AGE-RAGE interaction further exacerbates vascular permeability and promotes cellular dysfunction within the retinal microenvironment [4-7].

The inflammatory pathway is also triggered by hyperglycemia-induced oxidative imbalance, leading to elevated levels of pro-inflammatory cytokines such as interleukin-1β (IL-1β), tumor necrosis factor-alpha (TNF-α), and vascular adhesion molecules (VCAMs). These factors promote leukostasis and compromise the integrity of the blood-retinal barrier (BRB), resulting in increased vascular leakage and tissue injury. Another key driver of DR progression is angiogenesis, largely induced by hypoxia-driven overexpression of vascular endothelial growth factor (VEGF). VEGF promotes pathological neovascularization in advanced DR stages. However, the newly formed vessels are structurally unstable and prone to leakage and rupture, leading to complications such as hemorrhage, fibrosis, and retinal detachment, hallmarks of vision-threatening DR. Overall, these interconnected mechanisms form the basis of DR pathology and represent key therapeutic targets [4-7]. Multiple systemic and ocular factors influence DR development and progression. Prolonged diabetes duration, poor glycemic control, dyslipidemia, and renal dysfunction are key systemic contributors, while genetic predisposition, ethnicity, and associated comorbidities, such as cataracts, are the main ocular influencers [8-12].

Current DR treatments, including intravitreal anti-VEGF agents, corticosteroids, and laser photocoagulation, have long been used and represent a cornerstone of clinical management aimed at preserving visual function. Anti-VEGF agents effectively reduce macular edema and suppress neovascularization, corticosteroids help control intraocular inflammation, and laser photocoagulation remains valuable for ablating ischemic retinal regions to prevent further vascular damage. Although these therapies have demonstrated benefits in stabilizing advanced DR, they predominantly target late-stage pathological features rather than the early phases of the disease. Additionally, they are associated with notable drawbacks, such as the need for frequent intravitreal injections, an increased risk of complications like cataract formation and elevated intraocular pressure (IOP), and limited impact on the underlying systemic or molecular drivers of DR. These limitations emphasize the need for alternative therapeutic strategies aimed at earlier intervention and prevention of disease progression [13-16].

Metformin, a well-known anti-diabetic medication, is considered first-line treatment for type 2 diabetes (T2D), with established efficacy. Administered orally, metformin is absorbed mainly from the small intestine with a bioavailability of about 50-60%. It is not metabolized by the liver and is excreted unchanged by the kidneys, with an elimination half-life ranging from four to eight hours. The primary mechanism of action of metformin involves the activation of AMP-activated protein kinase (AMPK), a central regulator of cellular energy homeostasis, which subsequently leads to the reduction of hepatic gluconeogenesis and improved peripheral glucose uptake. The use of metformin is associated with minimal risk of hypoglycemia and modest weight reduction, thereby becoming a favorable choice in patients with insulin resistance and metabolic syndrome [17-19].

In addition to its classical metabolic actions, metformin exhibits a wide range of pleiotropic effects, including anti-inflammatory, antioxidant, anti-fibrotic, and immunomodulatory properties. These pleiotropic effects position metformin as a potential therapeutic agent across various medical fields, including cardiovascular diseases, polycystic ovary syndrome (PCOS), neurodegenerative disorders such as Alzheimer’s disease, and various malignancies. Metformin’s potential applications extend further to non-traditional fields, such as dentistry, in particular, in periodontal disease, which further reflects its broad therapeutic potential [20-25].

Recently, the investigation of metformin's role in DR treatment became an area of interest, as it was found to affect certain pathological mechanisms involved in DR. Many studies demonstrate its positive on DR development and progression. This narrative review explores the mechanisms through which metformin may exert its therapeutic effects in DR, discusses the clinical evidence, and identifies the limitations of metformin's potential use in DR treatment. 

Research methodology

While conducting this literature review, we performed an extensive search across various databases, including PubMed, Web of Science, Scopus, Cochrane Library, and Google Scholar, using a combination of MeSH terms and keywords such as "Metformin AND diabetic retinopathy", "Metformin AND oxidative stress AND inflammation in DR", and "Metformin AND angiogenesis in diabetic complications". Only peer-reviewed studies were included, with a primary focus on those published from 2020 onward.

## Review

Proposed mechanisms of metformin in DR

Antioxidant Effects

Metformin exerts its antioxidant effects through several interconnected mechanisms. At the mitochondrial level, it alleviates dysfunction by upregulating manganese superoxide dismutase (MnSOD) and enhancing the expression of peroxisome proliferator-activated receptor gamma coactivator 1-alpha (PGC-1α). Moreover, it attenuates the production of ROS induced by methylglyoxal and activates the nuclear factor erythroid 2-related factor 2 (Nrf2)/heme oxygenase-1 (HO-1) signaling pathway, a critical regulator of cellular antioxidant defense. Metformin also reduces levels of malondialdehyde (MDA), total oxidative status (TOS), and oxidative stress index (OSI), while simultaneously increasing total antioxidant capacity (TAC) [26-30]. Collectively, these actions contribute to the mitigation of oxidative damage within the retinal microenvironment in DR.

Anti-inflammatory Effects

Metformin has been shown to possess anti-inflammatory properties modulated by both AMPK-dependent and AMPK-independent mechanisms. Through the AMPK-dependent pathway, metformin inhibits inflammatory signaling cascades, including the TNF-α, nuclear factor kappa B (NF-κB), and mechanistic target of rapamycin (mTOR) pathways, while suppressing the expression of pro-inflammatory mediators such as intercellular adhesion molecule-1 (ICAM-1), IL-8, and monocyte chemoattractant protein-1 (MCP-1). The AMPK-independent pathway involves the activation of regulatory molecules, including Dicer, inducible 6-phosphofructo-2-kinase (iPFK2), and specific microRNAs (miRNAs), resulting in reduced cytokine expression. Furthermore, metformin has been demonstrated to inhibit NF-κB mRNA expression and prevent nuclear translocation of NF-κB p65, thereby downregulating inflammatory mediators. By attenuating IL-1 signaling and reducing ectopic lipid accumulation, metformin exerts multifaceted modulation of key inflammatory pathways implicated in DR [31-37].

Anti-angiogenic Effects

The effect of metformin on angiogenesis has been demonstrated in numerous studies; indeed, some suggest that part of metformin’s anticancer activity is attributed to its influence on angiogenesis. Metformin reduces VEGF through its action on the AMPK pathway, which in turn decreases endothelial cell proliferation and migration, consequently limiting abnormal neovascularization and vascular leakage. Additionally, metformin inhibits the expression of ICAM-1 and VCAM-1, thereby reducing endothelial dysfunction and leukocyte adhesion. By downregulating metalloproteinases (MMPs) and other adhesion molecules, metformin modulates the extracellular matrix and vascular permeability. It also decreases MTORC1 expression and enhances prolyl hydroxylases (PHDs), leading to reduced hypoxia-inducible factor-1 alpha (HIF-1-alpha) levels and, subsequently, attenuated hypoxia-driven inflammation and angiogenesis. Furthermore, some studies report that metformin affects cytoskeletal dynamics and endothelial migration via suppression of the actin-binding protein profilin-1 (PFN-1) [38-43].

Autophagy Modulation

Recent evidence indicates that metformin enhances autophagy and mitochondrial function by suppressing mitochondrial respiratory chain complex I (MRC-I), leading to a reduction in ATP production. The consequent decrease in ATP elevates AMP levels, which activate AMPK [44,45]. Activated AMPK initiates multiple downstream pathways that promote autophagy, with Unc-51-like autophagy-activating kinase 1 (ULK1) as a principal target. AMPK activates ULK1 via two mechanisms: direct phosphorylation of ULK1 and inhibition of mTORC1, a major negative regulator of autophagy [46,47]. AMPK suppresses mTORC1 by inactivating tuberous sclerosis complex 2 (TSC2), an essential activator of mTOR, and by directly phosphorylating raptor, a critical component of mTORC1. This disruption prevents the interaction between raptor and ULK1, thereby further promoting autophagy [47].

Regulation of Advanced Glycation End Products

Metformin’s effects on AGEs involve both reducing their synthesis and enhancing their clearance. It primarily lowers AGE synthesis by decreasing blood glucose levels, directly affecting the glycation process [48,49]. Metformin also interferes with α-dicarbonyl compounds, suppressing the Maillard reaction, a key pathway for AGE formation [50,51]. Beyond reducing AGE formation, metformin promotes their clearance by enhancing autophagy [50]. Additionally, it downregulates the RAGE, thereby attenuating inflammatory responses and oxidative stress associated with AGE accumulation [52-54]. Through these mechanisms, metformin mitigates AGE formation and its downstream pathological effects on the retinal vasculature.

Neuroprotective Effects

Metformin’s neuroprotective effects involve several mechanisms, many overlapping with its anti-inflammatory, antioxidant, autophagy-promoting, and anti-angiogenic actions. It enhances mitochondrial biogenesis, induces mitophagy, improves retinal structure, regulates neurotrophic factors, and supports Müller cell function, collectively contributing to its neuroprotective potential in DR.

Metformin promotes mitochondrial biogenesis primarily via AMPK activation, which phosphorylates PGC-1α and enhances SIRT1-mediated deacetylation, resulting in increased mitochondrial gene expression [55,56]. It also triggers mitophagy through multiple pathways, including PTEN-induced kinase 1 (PINK1), E3 ubiquitin-protein ligase parkin, BCL2/adenovirus E1B 19kDa-interacting protein 3 (BNIP3), NIP3-like protein X (Nix), and FUN14 domain-containing protein 1 (FUNDC1)-mediated mechanisms. Notably, FUNDC1-mediated mitophagy is facilitated by AMPK-regulated ULK1 phosphorylation [56-58].

Beyond promoting biogenesis, metformin upregulates brain-derived neurotrophic factor (BDNF), a key neurotrophic factor, by enhancing histone acetylation at its promoter region. This is mediated by AMPK activation and cyclic adenosine monophosphate-response element-binding protein (CREB) signaling, both essential for neuroprotection [21,59].

**Figure 1:**
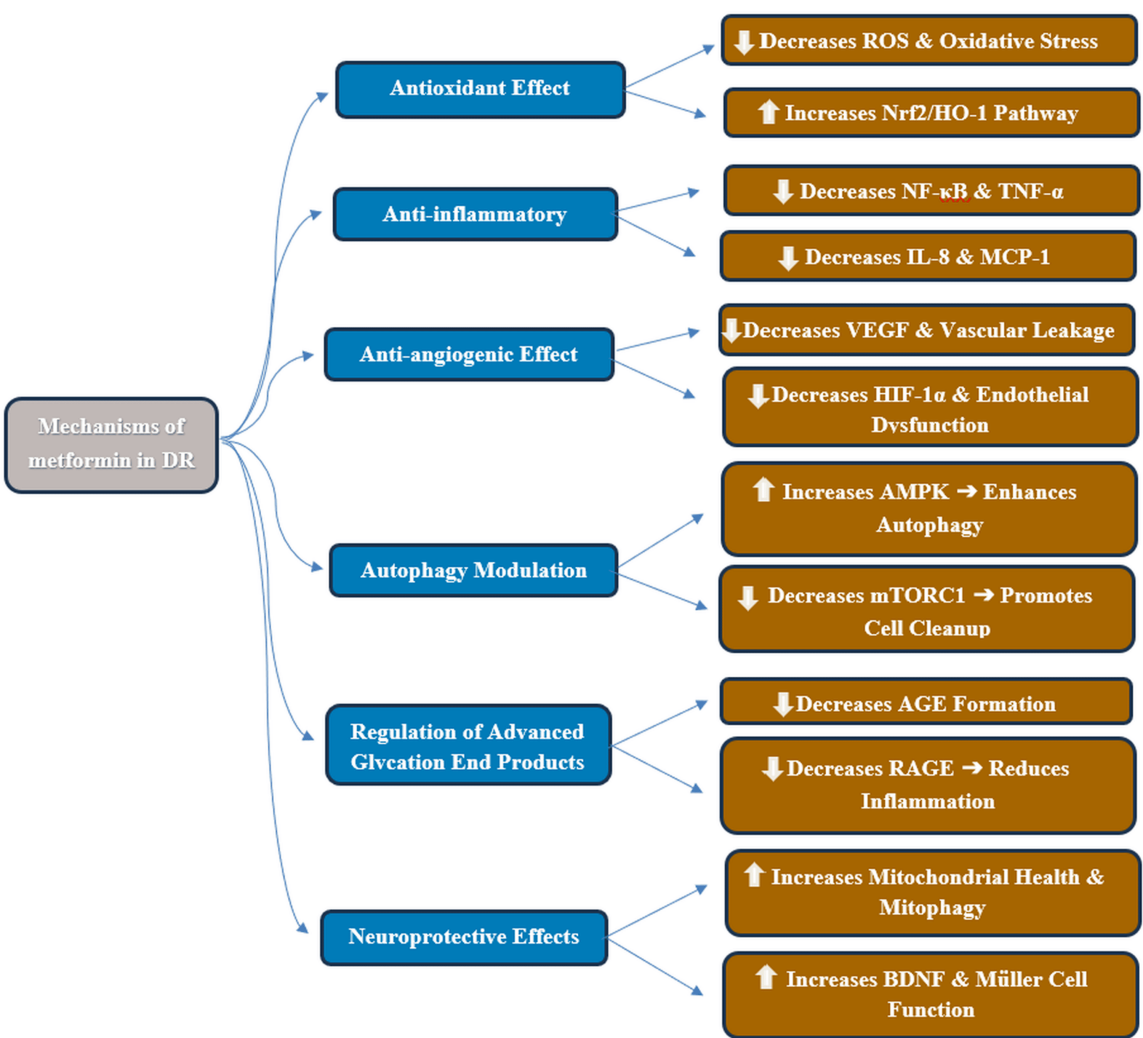
Algorithm of the proposed mechanisms of metformin in diabetic retinopathy. Abbreviations: ROS, reactive oxygen species; Nrf2, nuclear factor erythroid 2-related factor 2; HO-1, heme oxygenase-1; NF-κB, nuclear factor-kappa B; TNF-α, tumor necrosis factor-alpha; IL-8, interleukin-8; MCP-1, monocyte chemoattractant protein-1; VEGF, vascular endothelial growth factor; HIF-1α, hypoxia-inducible factor 1-alpha; AMPK, AMP-activated protein kinase; mTORC1, mechanistic target of rapamycin complex 1; AGE, advanced glycation end products; RAGE, receptor for advanced glycation end products; BDNF, brain-derived neurotrophic factor. This information is cited from references [26-64].

Additionally, metformin increases the expression and distribution of potassium inwardly rectifying channel subfamily J member 4 (Kir4.1), a channel critical for regulating extracellular potassium levels and preventing glutamate excitotoxicity, thereby supporting Müller cell function [60-62]. Furthermore, metformin ameliorates histopathological changes associated with DR, resulting in a more organized retinal cellular architecture and increased ganglion cell numbers [63,64]. The algorithm shown in Figure 1 summarizes the various physiological effects of metformin in DR.

Preclinical and clinical evidence

Reduction in the Risk of DR Development

Recent emerging evidence suggests that it may have protective effects for retinal/posterior segment diseases, including DR, age-related macular degeneration (AMD), inherited retinal degeneration such as retinitis pigmentosa (RP), primary open-angle glaucoma (POAG), retinal vein occlusion (RVO), and uveitis [65]. In preclinical mouse models where DR is induced by alloxan, Zhang et al. studied the effects of metformin on the development of DR as well as the mechanisms. Metformin was found to inhibit the VEGF-A protein translation by inducing a VEGF-A-targeting microRNA, microRNA-497a-5p, resulting in reduced retina neovascularization, which suggests the role of metformin in the prevention of the development of DR and progression by inhibiting pathological angiogenesis, a hallmark of the disease [66]. In vitro studies performed by Silva et al. involved human microvascular endothelial cells treated with metformin further showed that metformin significantly inhibited vessel formation and migration, significantly reduced collagen formation as evidenced by histological staining, improved endothelial dysfunction, and suppressed aberrant neovascularization [38].

Additionally, a clinical cross-sectional study by Mohammed et al. in the Diabetic Center in Karbala, Iraq, involving 522 patients (356 on insulin, 70 on metformin, and 96 healthy controls) assessed the effects of insulin versus metformin on key inflammatory markers and related complications. The results indicated that metformin users had markedly lower incidences of retinopathy (11.4% vs. 64.3%) and neuropathy (11.4% vs. 69.9%) compared to the insulin group, supporting its role in reducing the risk of microvascular complications, including DR [67]. While the duration of diabetes is a well-established risk factor for microvascular complications, the authors reported that it did not significantly influence any of the inflammatory markers studied, including E-selectin, which is strongly implicated in the progression of DR. This suggests that the observed differences in inflammatory markers and complication profiles were more closely related to the type of treatment rather than the duration of diabetes [67].

Reduction in DR Progression

The impact of metformin on DR progression has been investigated in both clinical and preclinical studies. A retrospective study by Li et al. involving 335 DR patients with T2D for ≥ 15 years aimed to evaluate the effects of long-term metformin on the severity of DR in high-risk T2D patients. The results demonstrated that long-term metformin use was associated with a significant reduction in DR severity compared to non-users where severe non-proliferative diabetic retinopathy (SNPDR) or proliferative diabetic retinopathy (PDR) was more often diagnosed in non-metformin users (67/142, 47%) versus metformin users (48/193, 25%) (p < 0.001), regardless of gender and race of the patients [68].

Consistent with these findings, a population-based cohort study by Fan et al. involved the analysis of 10,044 diabetic patients using data from the National Health Insurance Research Database to assess the relationship between metformin use and the severity of DR in patients with T2D. The authors concluded that metformin use correlated with a decreased risk of non-proliferative diabetic retinopathy (NPDR) and sight-threatening diabetic retinopathy (STDR) [69].

Combining metformin with other therapies, such as anti-VEGF agents, has also shown promising effects in reducing DR-related complications. A retrospective study by Uwimana et al. involving 109 patients with diabetic macular edema (DME) assessed the impact of concurrent metformin use on the efficacy of anti-VEGF therapy. All participants had a central retinal thickness (CRT) of ≥250 μm and received at least three intravitreal anti-VEGF injections between January 2020 and December 2021. Treatment resistance was defined as a CRT reduction of ≤25% following the initial injection series. Patients receiving metformin exhibited a marked anatomical improvement, with CRT decreasing from 415.64 ± 144.26 μm to 277.11 ± 99.25 μm, a 31.51% reduction. In contrast, the non-metformin group showed a smaller reduction, from 344.88 ± 129.48 μm to 318.29 ± 123.23 μm, equating to a 20.85% change. Additionally, resistance to anti-VEGF therapy was notably less frequent among metformin users (45.3%) compared to non-users (73.2%). While both groups demonstrated comparable gains in best-corrected visual acuity, these findings suggest that metformin may augment the anatomical response to anti-VEGF agents and potentially mitigate resistance, supporting its adjunctive role in managing DME and slowing DR progression [70].

Improvement of Retinal Microvascular Health

Metformin has been found to improve retinal microvascular health by modulating vascular inflammation, insulin sensitivity, mitochondrial function, and oxidative stress. In high-fat diet (HFD) animal models, this has been investigated in detail to understand metformin’s early vascular effects. In a controlled experiment, adult male rats were fed either standard chow or an HFD for two or four weeks. A subset of HFD-fed rats received oral metformin (300 mg/kg/day) throughout the feeding period. To evaluate insulin’s vascular and metabolic actions, a combination of the euglycemic-hyperinsulinemic clamp and contrast-enhanced ultrasound was used. HFD feeding led to increased adiposity without significant weight gain, accompanied by a marked reduction in insulin-stimulated glucose uptake and a complete loss of insulin-induced microvascular recruitment in skeletal muscle. In contrast, rats treated with metformin retained insulin-mediated microvascular responses by week two, and by week four, glucose disposal rates had returned to control levels. These changes were associated with reduced endothelial oxidative stress and vascular inflammation, along with improved insulin signaling within the endothelium, highlighting metformin's potential in diminishing oxidative stress and inflammation and preserving microvascular integrity [71].

The vascular benefits of metformin have also been observed in human studies. Liu et al. conducted a crossover study evaluating metformin’s effects in 11 subjects with nondiabetic metabolic syndrome who underwent 12 weeks of treatment with metformin or placebo, separated by an eight-week washout period. Large artery function was assessed using pulse wave velocity (PWV), radial pulse wave separation analysis (PWSA), and flow-mediated dilation (FMD), while muscle microvascular perfusion was measured by contrast-enhanced ultrasound during a euglycemic-hyperinsulinemic clamp. Metformin treatment significantly reduced body mass index, fat mass, and percentage of body fat, and improved systemic insulin sensitivity as indicated by higher glucose infusion rates during the clamp. Although metformin did not alter aortic stiffness (PWV) or brachial artery endothelial function (FMD), it enhanced radial pulse wave properties consistent with improved relaxation of small arterioles. Importantly, while both groups experienced a reduction in muscle microvascular blood volume in response to insulin, the decline was less pronounced in the metformin-treated group, indicating enhanced microvascular insulin sensitivity and function [72].

In addition, a cross-sectional investigation conducted in Spain by de Marañón et al. evaluated the impact of metformin on mitochondrial function in peripheral blood mononuclear cells (PBMCs) from a total of 242 participants. The cohort comprised 101 healthy volunteers, 93 individuals with type T2D undergoing metformin treatment, and 48 diabetic patients without metformin therapy. Results demonstrated that PBMCs from untreated diabetic subjects exhibited elevated ROS generation alongside a diminished expression of electron transport chain (ETC) complexes when compared to both healthy controls and those treated with metformin. Furthermore, critical mitophagy-related proteins, including PINK1 and parkin, as well as the mitochondrial biogenesis factor PGC1α, were markedly decreased in untreated T2D patients but showed recovery following metformin administration. Phosphorylation levels of AMPK, which were reduced in diabetes, were restored by metformin treatment. The study also noted that pro-inflammatory cytokines TNFα and IL-6, which were increased in untreated diabetic subjects, were significantly lowered after metformin therapy. Collectively, these findings suggest that metformin enhances mitochondrial function by mitigating oxidative stress, promoting mitophagy, and activating AMPK pathways, thereby potentially preserving mitochondrial health and microvascular function [56].

Protective Role Against DR Complications

The protective effect of metformin against DR complications has been evaluated in preclinical models. Metformin was shown to preserve retinal pigment epithelial (RPE) cell function in both in vivo and in vitro studies, suggesting its role in maintaining retinal health [73]. Additionally, in an oxygen-induced retinopathy mouse model, metformin reduced pathological angiogenesis by decreasing neovascular tufts and vascular overgrowth. The study also observed diminished phosphorylated ribosomal protein S6 (pS6) immunoreactivity in vascular cells, a marker of mTORC1 activation, indicating that metformin directly targets angiogenesis in advanced DR independently of VEGF and VEGFR2 expression [74].

In clinical studies, a retrospective chart review of 335 DR patients by Li et al. assessed the long-term impact of metformin on DR severity. The prevalence of severe NPDR or PDR was significantly lower in metformin users compared to non-users (25% vs. 47%) [68]. Moreover, a large-scale population-based cohort study by Fan et al. involving 10,044 individuals examined the association between metformin use and DR severity in patients with T2D. The study found that metformin use was significantly associated with a reduced risk of developing STDR, with an adjusted hazard ratio (aHR) of 0.29 (95% CI, 0.19-0.45) compared to non-users. Furthermore, among patients with existing NPDR, metformin use correlated with a lower likelihood of progression to STDR (aHR = 0.54, 95% CI, 0.28-1.01) [69]. Interestingly, Li et al. revealed that patients receiving metformin at a cumulative defined daily dose (DDD) of less than 30 had a significantly lower risk of developing DR (aHR = 0.77; 95% CI: 0.60-0.98). However, those with higher cumulative metformin exposure, particularly at doses exceeding 25 DDDs, demonstrated a 2.43-fold increased risk of DR (95% CI: 1.37-4.30) compared to patients treated with sulfonylureas, highlighting a potential dose-dependent paradoxical effect of metformin on DR development [75].

These findings highlight metformin’s dual role in preventing the onset of advanced DR and slowing its progression in affected patients. Collectively, these data underscore the multifaceted role of metformin in protecting against DR complications. Table 1 summarizes the preclinical and clinical benefits of metformin and their key findings.

**Table 1:** Preclinical and clinical benefits of metformin in diabetic retinopathy. Abbreviations: DR, diabetic retinopathy; NPDR, non-proliferative diabetic retinopathy; STDR, sight-threatening diabetic retinopathy; PDR, proliferative diabetic retinopathy; VEGF, vascular endothelial growth factor; AMPK, AMP-activated protein kinase; eNOS, endothelial nitric oxide synthase; RPE, retinal pigment epithelium; ROS, reactive oxygen species; T2D, type 2 diabetes; mTORC1, mechanistic target of rapamycin complex 1; pS6, phosphorylated ribosomal protein S6; aHR, adjusted hazard ratio.

Preclinical and clinical benefits of using metformin	Focus	Key findings	References
Reduction in the risk of DR development	Preventive effects of metformin	Inhibits pathological angiogenesis. Improves endothelial function. Lower DR (11.4%) and neuropathy (11.4%) in metformin users vs. insulin users (64.3%, 69.9%).	[38,65-67]
Reduction in DR progression	Slowing disease severity	Long-term use is linked with reduced DR severity. Lower risk of NPDR & STDR in a large cohort (n = 10,044). Reduces PDR/NPDR and neovascularization. Reduces DR complications when combined with anti-VEGF therapies.	[68-70]
Improvement of retinal microvascular health	Mechanisms at the vascular level	Reduces oxidative stress, and improves endothelial function via AMPK & eNOS. Enhances vasodilation. Reduces inflammation (NF-κB p65 DNA-binding activity). Increase insulin microvascular responses. Lowers mitochondrial ROS in T2D patients.	[56,71,72]
Protective role against DR complications	Protective effect against advanced DR	Preserves RPE cell function in vivo/in vitro. Reduces pathological angiogenesis via the mTORC1 pathway (pS6). Lower severe NPDR/PDR prevalence in metformin users (25% vs. 47%). Reduced risk of STDR (aHR: 0.29); slows NPDR progression to STDR (aHR: 0.54).	[68,69,73-75]

Metformin versus conventional therapies in DR

Existing therapies for DR primarily focus on addressing advanced stages of the disease and their complications. Standard interventions such as laser photocoagulation, anti-VEGF agents, or corticosteroids have proven effective in limiting vision loss and controlling pathological retinal neovascularization. Nonetheless, the invasive nature and possible adverse effects, including retinal scarring, increased IOP, and the need for repeated treatments, should be taken into consideration [13-16].

In contrast, metformin has a long-established safety and tolerability profile through decades of clinical use. Its widespread availability and low cost may make it an attractive option, particularly in resource-limited settings. Furthermore, metformin’s pleiotropic properties may offer additional benefits to patients with diabetic complications, potentially improving overall vascular health and metabolic balance. Of particular interest, metformin may also influence the early stages of DR, thereby helping to prevent disease progression [17-19].

Combination effects and comparative safety of metformin with antidiabetic agents in DR

Combination therapy involving metformin and other antidiabetic agents demonstrates variable effects on the progression of DR in patients with T2D. While metformin monotherapy is associated with a reduced risk of both NPDR and STDR, combination with dipeptidyl peptidase-4 inhibitor (DPP-4I) confers an enhanced protective effect against NPDR development, particularly when initiated early in the disease course and in patients with lower adapted Diabetes Complications Severity Index (aDCSI) scores [69]. Similarly, adjunctive therapy with sodium-glucose co-transporter 2 inhibitors (SGLT2is) has been shown, in a large Taiwanese cohort, to modestly but significantly reduce the risk of DR progression (aHR = 0.89, 95% CI: 0.81-0.99) after controlling for demographic, clinical, and treatment-related confounders [76]. Notably, the duration of SGLT2is use is critical; short-term exposure (<2 years) correlates with an increased risk of DR progression, whereas prolonged use beyond two years markedly reduces this risk (aHR = 0.41, 95% CI: 0.35-0.48; P < 0.001), a finding supported by ophthalmologic sensitivity analyses [76]. In contrast, the addition of sulfonylureas to metformin appears to increase the risk of DR progression [69]. These data collectively highlight that the impact of metformin-based combination therapies on retinal outcomes is heterogeneous, underscoring the importance of drug choice and treatment duration in optimizing DR management.

In parallel, the combination therapy of metformin with other antidiabetic agents also demonstrates a favorable safety profile in the context of DR. While most systemic glucose-lowering therapies are not specifically designed for DR management, their long-term metabolic effects can influence microvascular outcomes. A recent umbrella review and meta-analysis by Yang et al. evaluated the association between various antidiabetic drugs and DR risk in patients with T2D. The study found that metformin, together with SGLT2is, GLP-1 receptor agonists, and DPP-4I, was not significantly associated with increased DR risk. Conversely, insulin use was linked to a significantly higher risk of DR progression (odds ratio (OR): 2.47, 95% CI: 2.04-2.99). These findings further support the safety of metformin-based combination therapies and reinforce their role in early-stage DR treatment strategies [77].

Challenges and limitations

Limitations to the optimal therapeutic efficacy of metformin in DR include the absence of randomized controlled trials validating current findings, which primarily remain observational in nature, carrying a risk of bias due to confounding variables, selection bias, and non-standardized protocols, heterogeneous responses across different DR stages, safety considerations in patients with renal impairment, as those with preserved renal function may particularly benefit from metformin due to the reduced risk of lactic acidosis, a known concern in individuals with compromised renal parameters, and the potential for adverse effects (e.g., vitamin B12 deficiency) and drug-drug interactions [17,68]. If these limitations are addressed, metformin could emerge as a more effective and safer option for managing DR.

Future directions

For the successful implementation of metformin in DR treatment, future studies should focus on determining the optimal dosing and duration of therapy specifically for retinal protection. Randomized controlled trials are also needed to evaluate metformin as an adjunct to current DR therapies, potentially clarifying its synergistic benefits in advanced stages. Moreover, the development and refinement of novel metformin formulations, such as ocular-targeted delivery systems including intravitreal injections, sustained-release implants, or nanoparticle-based carriers, hold significant promise.

These innovative delivery methods aim to maximize local drug concentration within retinal tissues, thereby enhancing therapeutic efficacy while substantially reducing systemic exposure and associated side effects. Addressing pharmacokinetic challenges and optimizing formulation stability will be critical in advancing these approaches. To achieve a comprehensive understanding of metformin’s long-term effects, cohort studies are warranted to assess its impact on visual outcomes and overall treatment burden.

## Conclusions

Emerging evidence from both preclinical and clinical studies highlights metformin’s potential in the treatment of DR through its multifaceted actions on oxidative stress, inflammation, and pathological angiogenesis. Metformin use has been associated with improvement in retinal microvascular health, reduction in DR development and progression, as well as a protective role against DR-related complications.

Although this growing evidence is promising, the potential adverse effects, safety concerns, especially in patients with renal impairment, and the lack of large-scale studies supporting this evidence remain a barrier. Establishing optimal dosing protocols, assessing long-term safety, and exploring combination therapies in future research is essential to fully realize metformin’s potential in DR management.
